# Supplementary material for: Smooth Interpolating Curves with Local Control and Monotone Alternating Curvature
Source: Comput Graph Forum. 2022 Oct 6;41(5):25–38. doi: 10.1111/cgf.14600 (PMC9827861; doi:10.1111/cgf.14600)
Supplement: Supplementary file 1 — Supplement Material [file CGF-41-25-s001.zip › Local-Smooth-Interpolating-MonoCurvature/extern/clothoids/submodules/Utils/docs/api-cpp/program_listing_file_Utils_Numbers.hxx.html]

Program Listing for File Numbers.hxx — Utils v1.2

### Navigation

- index
- toc
- Utils »
- Program Listing for File Numbers.hxx

# Program Listing for File Numbers.hxx¶

↰ Return to documentation for file (`Utils/Numbers.hxx`)

```
/*--------------------------------------------------------------------------*\
 |                                                                          |
 |  Copyright (C) 2020                                                      |
 |                                                                          |
 |         , __                 , __                                        |
 |        /|/  \               /|/  \                                       |
 |         | __/ _   ,_         | __/ _   ,_                                |
 |         |   \|/  /  |  |   | |   \|/  /  |  |   |                        |
 |         |(__/|__/   |_/ \_/|/|(__/|__/   |_/ \_/|/                       |
 |                           /|                   /|                        |
 |                           \|                   \|                        |
 |                                                                          |
 |      Enrico Bertolazzi                                                   |
 |      Dipartimento di Ingegneria Industriale                              |
 |      Universita` degli Studi di Trento                                   |
 |      email: enrico.bertolazzi@unitn.it                                   |
 |                                                                          |
\*--------------------------------------------------------------------------*/


#pragma once

#ifndef DOXYGEN_SHOULD_SKIP_THIS
#ifndef UTILS_NUMBERS_dot_HXX
#define UTILS_NUMBERS_dot_HXX
#endif

namespace Utils {

  #ifndef DOXYGEN_SHOULD_SKIP_THIS
  using std::numeric_limits;
  using std::string;
  using std::sqrt;
  using std::fpclassify;
  using std::floor;
  #endif

  /*
  //    ____                _              _
  //   / ___|___  _ __  ___| |_ __ _ _ __ | |_ ___
  //  | |   / _ \| '_ \/ __| __/ _` | '_ \| __/ __|
  //  | |__| (_) | | | \__ \ || (_| | | | | |_\__ \
  //   \____\___/|_| |_|___/\__\__,_|_| |_|\__|___/
  */

  template <typename T> T NaN();
  template <typename T> T Inf();
  template <typename T> T machineEps();
  template <typename T> T sqrtMachineEps();
  template <typename T> T maximumValue();
  template <typename T> T minimumValue();

  #ifndef DOXYGEN_SHOULD_SKIP_THIS
  template <> inline float
  NaN() { return numeric_limits<float>::quiet_NaN(); }

  template <> inline double
  NaN() { return numeric_limits<double>::quiet_NaN(); }

  template <> inline float
  Inf() { return numeric_limits<float>::infinity(); }

  template <> inline double
  Inf() { return numeric_limits<double>::infinity(); }

  template <> inline float
  machineEps() { return numeric_limits<float>::epsilon(); }

  template <> inline double
  machineEps() { return numeric_limits<double>::epsilon(); }

  template <> inline float
  sqrtMachineEps() { return sqrt(numeric_limits<float>::epsilon()); }

  template <> inline double
  sqrtMachineEps() { return sqrt(numeric_limits<double>::epsilon()); }

  template <> inline float
  maximumValue() { return sqrt(numeric_limits<float>::max()); }

  template <> inline double
  maximumValue() { return sqrt(numeric_limits<double>::max()); }

  template <> inline float
  minimumValue() { return sqrt(numeric_limits<float>::min()); }

  template <> inline double
  minimumValue() { return sqrt(numeric_limits<double>::min()); }
  #endif

  static
  inline
  bool isZero( double x )
  { return FP_ZERO == fpclassify(x); }

  static
  inline
  bool isZero( float x )
  { return FP_ZERO == fpclassify(x); }

  static
  inline
  bool isInfinite( double x )
  { return FP_INFINITE == fpclassify(x); }

  static
  inline
  bool isInfinite( float x )
  { return FP_INFINITE == fpclassify(x); }

  static
  inline
  bool isNaN( double x )
  { return FP_NAN == fpclassify(x); }

  static
  inline
  bool isNaN( float x )
  { return FP_NAN == fpclassify(x); }

  static
  inline
  bool isRegular( double x )
  { return !( FP_INFINITE == fpclassify(x) ||
              FP_NAN      == fpclassify(x) ); }

  static
  inline
  bool isRegular( float x )
  { return !( FP_INFINITE == fpclassify(x) ||
              FP_NAN      == fpclassify(x) ); }

  // added alias
  static
  inline
  bool isFinite( double x )
  { return !( FP_INFINITE == fpclassify(x) ||
              FP_NAN      == fpclassify(x) ); }

  static
  inline
  bool isFinite( float x )
  { return !( FP_INFINITE == fpclassify(x) ||
              FP_NAN      == fpclassify(x) ); }

  static
  inline
  bool isInteger( double x )
  { return isZero( x-static_cast<long>(floor(x)) ); }

  static
  inline
  bool isInteger( float x )
  { return isZero( x-static_cast<long>(floor(x)) ); }

  static
  inline
  bool isUnsigned( double x ) { return isInteger(x) && x >= 0; }

  static
  inline
  bool isUnsigned( float x ) { return isInteger(x) && x >= 0; }

  //============================================================================

  bool
  foundNaN( double const * const pv, int DIM );

  bool
  foundNaN( float const * const pv, int DIM );

  void
  checkNaN(
    double const * const pv,
    char   const * const v_name,
    int                  DIM,
    int                  line,
    char   const * const file
  );

  void
  checkNaN(
    float const * const pv,
    char  const * const v_name,
    int                 DIM,
    int                 line,
    char  const * const file
  );

  //============================================================================

  static double const m_e = 2.718281828459045235360287471352662497757;

  static double const m_pi = 3.141592653589793238462643383279502884197;

  static double const m_2pi = 6.283185307179586476925286766559005768394;

  static double const m_pi_2 = 1.570796326794896619231321691639751442098;

  static double const m_pi_4 = 0.7853981633974483096156608458198757210492;

  static double const m_1_pi = 0.3183098861837906715377675267450287240689;

  static double const m_2_pi = 0.6366197723675813430755350534900574481378;

  static double const m_sqrtpi = 1.772453850905516027298167483341145182798;

  static double const m_2_sqrtpi = 1.128379167095512573896158903121545171688;

  static double const m_sqrt2 = 1.414213562373095048801688724209698078570;

  static double const m_1_sqrt2 = 0.7071067811865475244008443621048490392850;

}

#endif
```

### Quick search

### Table of Contents

- rang - Colors for your Terminal
- Zstr
- C++ API

«
hide menu

menu
sidebar
»

### Navigation

- index
- toc
- Utils »
- Program Listing for File Numbers.hxx

© Copyright 2021, Enrico Bertolazzi.
Created using Sphinx 4.2.0.
